# Effect of a patient-support program on once-daily teriparatide adherence and persistence in the Japan Fracture Observational Study (JFOS)

**DOI:** 10.1007/s11657-018-0487-8

**Published:** 2018-07-05

**Authors:** Masayo Sato, Mika Tsujimoto, Kenta Kajimoto, Hideyuki Uetake, Hiroo Shimoda, Saeko Fujiwara

**Affiliations:** 1 Medicines Development Unit Japan, Eli Lilly Japan K.K, Kobe, Hyogo Japan; 2 Statistical Analysis Department, CDM Division, CMIC Co., Ltd., Tokyo, Japan; 3 Health Management and Promotion Center, Hiroshima Atomic Bomb Casualty Council, Hiroshima, Japan

**Keywords:** Adherence, Japan, Osteoporosis, Patient-support program, Persistence, Teriparatide

## Abstract

***Summary*:**

Japanese patients with osteoporosis prescribed once-daily teriparatide for 24 months could enroll in a patient-support program designed to aid adherence and persistence. Patients enrolled in the program had higher adherence and persistence rates than those who did not enroll, highlighting the value of patient-support programs for improving adherence and persistence.

**Objective:**

To assess the effect of a patient-support program on once-daily teriparatide adherence and persistence of patients who did and did not enroll.

**Methods:**

In the 24-month Japan Fracture Observational Study, patients with osteoporosis prescribed teriparatide 20 μg/day (*N* = 1996) could freely enroll in a patient-support program (call center support, monthly calendar, certificates of recognition). Outcome measures were medication adherence (investigator assessed) and persistence (first date of teriparatide use to last date of use or study end). Multivariate logistic models were applied for adherence, and Kaplan-Meier survival curve for persistence.

**Results:**

Overall, mean ± standard deviation (SD) age was 76.9 ± 7.9 years, and the proportion of female patients was 90.1%. Program enrollment status was 39.6% yes (*n* = 790), 22.9% no (*n* = 458), and 37.5% unknown (*n* = 748). In the analysis sample (1248 patients), adherence (> 75%) to teriparatide was more likely for patients enrolled in the support program (54.2 vs. 48.3%; adjusted odds ratio 1.44 [95% confidence intervals 1.04–2.00], *p* = 0.030). Good to very good (> 75%) adherence was also associated with smoking (negative association) and previous osteoporosis therapy (marginal positive association). Persistence rates were greater for patients enrolled in the support program than not enrolled (12 months 77.2 vs. 69.6%; 24 months 63.2 vs. 54.8%).

**Conclusions:**

Once-daily teriparatide adherence and persistence rates were higher among patients who enrolled in a patient-support program than among those who did not, highlighting the value of patient-support programs for improving adherence and persistence.

## Introduction

Osteoporotic fractures are a major public health burden in Japan’s “super-aged” society; the percentage of the Japanese population aged ≥ 65 years reached 25% in 2013 and is anticipated to exceed 30% in 2025 [1]. Osteoporotic fractures mostly (≥ 85%) result from falls [1] and cause significant disability that necessitates care (e.g., medication, nursing, rehabilitation, surgery) at a substantial cost [2, 3]. In Japan, the primary reason elderly people require long-term care insurance is falls and fall-related fractures [4]; the number of people who require long-term care insurance has doubled since the service was introduced in 2000 [5].

Osteoporosis medication can reduce osteoporotic fracture risk [6]. However, low adherence and persistence with these medications are common, reducing their clinical effectiveness and cost-effectiveness [7]. Osteoporosis medication non-adherence (medication possession ratio [MPR] < 80%) and non-persistence (> 30 days’ treatment gap) have been found to increase the risk of osteoporotic fracture by 28 and 32%, respectively [6]. As a consequence, osteoporosis medication non-adherence has been estimated to double the cost per quality-adjusted life-year gained [8].

Teriparatide (recombinant human parathyroid hormone), an anabolic agent that stimulates osteoblastic bone formation, has been shown to improve skeletal health and outcomes when administered continuously for 24 months rather than for shorter periods [9]. Medical and pharmacy claims database studies of once-daily teriparatide use in the USA [10, 11], Japan [12], and Taiwan [13] have also indicated that higher rates of adherence and persistence were associated with reduced fracture risk and, in the Japan study, reduced hospital admissions and inpatient costs [12].

Strategies to help improve medication adherence and persistence include patient-support programs that consist of interventions such as individualized medication counseling, call center support, and/or virtual reminders to take medication. Patient-support programs to help patients administer once-daily teriparatide and provide follow-up support have been of benefit for European patients, with reported persistence rates considered high, ranging from 64.1% (*N* = 23,069) at 24 months [14] to 81.5% (*N* = 5413) over 15 months [15] and 85.6% (*N* = 382) at 18 months [16]. However, the effect of a patient-support program on once-daily teriparatide adherence and persistence for patients with osteoporosis in Japan has not been evaluated.

The Japan Fracture Observational Study (JFOS) was a prospective observational study that assessed the effectiveness of once-daily teriparatide in Japanese patients with osteoporosis treated in a real-world clinical setting. The baseline patient characteristics, preliminary 12-month findings [17], and final 24-month findings for JFOS have been published [18]. The main aim of this secondary analysis of the original study was to assess the effect of patient-support program enrollment on adherence to once-daily teriparatide. In addition, we reported 12-month and 24-month persistence with once-daily teriparatide for patients who did and did not enroll in the program.

## Materials and methods

### Study design and population

The study design and characteristics of patients participating in JFOS have been described previously [17, 18]. In brief, JFOS was a multicenter, prospective, observational study of Japanese patients with osteoporosis at high risk of clinical fracture from 175 hospitals and clinics including departments of orthopedic surgery, internal medicine, and gynecology. Patients were prescribed teriparatide 20 μg once daily by subcutaneous injection as part of their routine clinical care. Decisions regarding patient care were at the discretion of the participating study physicians. Patients gave written consent before enrollment and could withdraw from the study at any time without consequence. The first patient was enrolled in October 2012, with the first patient visit on September 21, 2012, and follow-up of 24 months. The study was conducted in accordance with the Japanese guideline for Good Post-marketing Study Practice (by the Ministry of Health, Labor and Welfare Ordinance No. 171; 20 Dec. 2004) and applicable laws and regulations.

### Patient-support program

The patient-support program was designed to aid patient adherence and persistence with once-daily teriparatide treatment. The program was developed, funded, and supported by Eli Lilly Japan K.K. Patients were informed about the program by their physicians and/or medical staff and were provided a registration card. Patients could enroll in the program at any time and enrollment was voluntary and free; to enroll, patients were required to complete and mail the registration card. As the patient-support program was independent from this study, the participants’ personal information, including identification, was not available for inclusion in the analyses. As such, information on enrollment status was reported by investigators through communication with patients.

The patient-support program consisted of three components: (1) call center support, (2) a page-a-day monthly calendar, and (3) certificates of recognition. At the call center, personnel phoned patients, at their request, during the first 6 months of treatment to support patients with injecting teriparatide, and they received inbound calls from patients from 7 am to 10 pm every day to answer any questions related to teriparatide treatment. A calendar containing a daily injection checklist was sent to patients each month to remind patients to administer their daily injection and to enhance patient engagement with their treatment. Certificates of recognition were sent to patients when they maintained treatment at 3, 6, 12, and 18 months and completed the full course (24 months) of treatment.

### Outcome measures

Two different measures were used to assess adherence and persistence. The assessment of adherence was conducted by investigators at each visit (0, 3, 6, 12, 18, and 24 months) and was based on communication with patients and/or on an estimate of the volume of teriparatide remaining in each pen and the number of pens prescribed to determine how many injections a patient had self-administered between visits. Adherence was categorized by investigators at each visit as < 50% (poor), 50 to 75% (not so good), > 75 to 99% (good), or 100% (very good). Persistence was measured from the first date to the last date of teriparatide use or the end of the 24-month observation period.

### Statistical analysis

Overall patient demographics and clinical characteristics were assessed at baseline as described previously [17]. Patients were grouped according to patient-support program enrollment: yes (ever enrolled at any time during the 24-month observation period), no (did not enroll during the 24-month observation period), and unknown (enrollment status unknown).

In general, statistical tests were performed for exploratory purposes, and all *p* values should be considered as reference values at a significance level (α) of 0.05 for any inferences. Patient demographics and clinical characteristics were summarized using descriptive statistics and were compared between yes and no patients using a two-sample *t* test for continuous variables and Fisher’s exact test for categorical variables. Subsequent analyses, including bivariate and multivariate analysis as well as Kaplan-Meier evaluation, were conducted by excluding the unknown group, leaving a total yes (*n* = 790) and no (*n* = 458) population of 1248 patients. Good to very good (> 75%) adherence was summarized using descriptive statistics and was compared between yes and no patients using a bivariate (Fisher’s exact test) and a multivariate logistic regression model. The multivariate logistic regression model assessed the association between good to very good (> 75%) adherence and support program enrollment status, and was adjusted for covariates. As the first step, the model included the following variables: enrollment status of the patient-support program as the independent variable of interest, age, sex, body mass index (kg/m^2^), family history of hip fracture, smoking status, alcohol use, comorbidities, previous osteoporosis medications, concomitant corticosteroid administration, back pain using the visual analog scale (VAS) at baseline, and prevalent vertebral fractures (0, 1, ≥ 2) and prevalent non-vertebral fractures (yes/no). Covariates were then selected by the backward elimination method (criterion *p* < 0.10). Age and sex were kept in the model because of their clinical importance. Standardized bone mineral density (sBMD) was calculated according to Hui et al. [19] and Lu et al. [20]. Persistence with once-daily teriparatide was reported using Kaplan-Meier survival curves according to enrollment status of the patient-support program using start and end dates, with censoring if the investigator indicated that the patient discontinued treatment, was lost to follow-up, or completed 24 months. All variables were analyzed using SAS version 9.1 (SAS Institute Inc., Cary, NC, USA).

## Results

### Patient disposition and baseline demographics and clinical characteristics

Of the 1996 patients participating in JFOS, 790 (39.6%) were enrolled in the patient-support program, 458 (22.9%) did not enroll in the program, and 748 (37.5%) had unknown enrollment status. In the total population, mean ± standard deviation (SD) age was 76.9 ± 7.9 years, and 90.1% of patients were female.

There were several differences in baseline demographics and clinical characteristics between patients who enrolled in the support program and those who did not (Table 1). Compared with patients who did not enroll, significantly more patients who enrolled were female, had a family history of hip fracture, a higher rate of prevalent non-vertebral fractures, less severe back pain, and a higher previous use of osteoporosis medications, specifically bisphosphonates and active vitamin D_3_ (Table 1). Numerically, fewer patients in the enrolled group consumed alcohol, had comorbidities, and used concomitant corticosteroids, and numerically, more were current smokers. sBMD and T scores of the lumbar spine (L2-L4), sBMD and T scores of the total hip, and procollagen type 1 aminoterminal propeptide (P1NP) concentrations were similar between patients who were enrolled and those who were not (Table 1).

**Table 1:** Baseline demographics and clinical characteristics

Variable, *n* (%)	All	Patient-support program enrollment			
	*N* = 1996	Yes	No	Unknown	*p* value*
		*N* = 790	*N* = 458	*N* = 748	
Age (years), mean ± SD	76.9 ± 7.9	76.9 ± 7.8	77.0 ± 7.9	76.9 ± 7.8	0.928
Female sex	1798 (90.1)	730 (92.4)	406 (88.6)	662 (88.5)	0.031
BMI (kg/m^2^)					
*N*	1504	615	349	540	
Mean ± SD	21.8 ± 3.5	21.8 ± 3.6	21.8 ± 3.3	21.9 ± 3.6	0.902
Current smoker	84 (4.2)	39 (4.9)	17 (3.7)	28 (3.7)	0.322
Alcohol, ≥ 3 units consumed daily^a^	88 (4.4)	25 (3.2)	26 (5.7)	37 (4.9)	0.053
Family history of hip fracture	99 (5.0)	53 (6.7)	15 (3.3)	31 (4.1)	0.012
Comorbidities	510 (25.6)	185 (23.4)	113 (24.7)	212 (28.3)	0.836
Previous osteoporosis medications^b^	1023 (51.3)	485 (61.4)	235 (51.3)	303 (40.5)	< 0.001
Bisphosphonate	676 (33.9)	353 (44.7)	149 (32.5)	174 (23.3)	< 0.001
SERMs^c^	168 (8.4)	81 (10.3)	35 (7.6)	52 (7.0)	0.131
Active vitamin D_3_	347 (17.4)	165 (20.9)	69 (15.1)	113 (15.1)	0.013
Calcitonin	98 (4.9)	50 (6.3)	18 (3.9)	30 (4.0)	0.092
Calcium	78 (3.9)	27 (3.4)	23 (5.0)	28 (3.7)	0.179
Other	45 (2.3)	8 (1.0)	15 (3.3)	22 (2.9)	0.007
Concomitant corticosteroids	54 (2.7)	18 (2.3)	19 (4.1)	17 (2.3)	0.084
Prevalent vertebral fractures	1321 (66.2)	533 (67.5)	324 (70.7)	464 (62.0)	0.254
0	675 (33.8)	257 (32.5)	134 (29.3)	284 (38.0)	0.286
1	622 (31.2)	221 (28.0)	146 (31.9)	255 (34.1)	
≥ 2	699 (35.0)	312 (39.5)	178 (38.9)	209 (27.9)	
Prevalent non-vertebral fractures	419 (21.0)	199 (25.2)	73 (15.9)	147 (19.7)	< 0.001
Lumbar spine (L2-L4)					
sBMD (g/cm^2^)^d^					
*N*	680	266	157	257	
Mean ± SD	0.73 ± 0.15	0.73 ± 0.14	0.73 ± 0.17	0.72 ± 0.14	0.735
T score (SD)^e^					
*N*	677	266	157	254	0.660
Mean ± SD	− 2.83 ± 1.14	− 2.84 ± 1.10	− 2.79 ± 1.29	− 2.86 ± 1.08	
Total hip					
sBMD (g/cm^2^)^d^					
*N*	617	242	152	223	0.864
Mean ± SD	0.55 ± 0.12	0.55 ± 0.13	0.55 ± 0.11	0.54 ± 0.11	
T score^e^					
*N*	612	242	152	218	0.852
Mean ± SD	− 3.26 ± 1.18	− 3.19 ± 1.33	− 3.17 ± 1.03	− 3.39 ± 1.10	
P1NP^f^ (μg/L)					
*N*	1080	469	242	369	0.368
Mean ± SD	48.8 ± 31.4	46.5 ± 30.4	48.6 ± 29.0	52.0 ± 33.8	
Back pain (VAS, mm)					
*N*	1725	718	407	600	
Mean ± SD	50.7 ± 30.6	46.9 ± 30.6	55.8 ± 30.9	51.9 ± 29.8	< 0.001

*BMI* body mass index, *P1NP* procollagen type 1 aminoterminal propeptide, *sBMD* standardized bone mineral density, *SD* standard deviation, *SERM* selective estrogen receptor modulator, *VAS* visual analog scale

*p values are for differences in baseline demographics and clinical characteristics between yes and no patients and were calculated using a two-sample *t* test for continuous variables and Fisher’s exact test for categorical variables

^a^1 unit of alcohol was 285 mL of beer, 120 mL of wine, 30 mL of distilled spirits, or 60 mL of aperitifs

^b^Patients may have had > 1 comorbidity or previous osteoporosis medication

^c^SERMs included raloxifene hydrochloride and bazedoxifene acetate

^d^sBMD was calculated according to Hui et al. [19] and Lu et al. [20]

^e^Mean BMDs of young adult Japanese population [23] were used as reference values for T score calculations. Measurements obtained with the scanner of CooperSurgical Inc. (formerly Norland) product were not applied to SD calculation for male lumbar spine BMD, or for male or female total hip BMD because of unavailability of corresponding Japanese young adult men

^f^P1NP reference ranges were 17.1–64.7 μg/L for Japanese premenopausal women and 21.9–79.1 μg/L for postmenopausal women [24, 25]

### Adherence to once-daily teriparatide

A total of 1248 patients were included in the analysis of adherence. Among 790 patients enrolled in the patient-support program, 428 (54.2%) patients had good to very good (> 75%) adherence compared with 221 (48.3%) patients not enrolled in the program (Table 2). Patients enrolled in the patient-support program were 1.27 times more likely to have good to very good adherence compared with patients not enrolled (crude odds ratio [OR] 1.27 [95% confidence interval (CI) 1.01–1.60], *p* = 0.046). Of the 1248 patients, 655 patients had complete information on all variables and were included in the multivariate analysis. After adjusting for patient characteristics, patients who were enrolled in the patient-support program were 1.44 times more likely (adjusted OR 1.44 [95% CI 1.04–2.00], *p* = 0.030) to adhere to once-daily teriparatide than patients who were not enrolled. The likelihood of good to very good adherence was significantly higher for enrolled patients than those who did not enroll, even after adjustment for covariates (Table 2). Covariates included in the final multivariate regression model were age, sex, smoking status, and use of previous osteoporosis medication. All variables included were identified by the backward elimination method. Smoking was negatively associated with adherence (OR 0.31 [95% CI 0.14–0.68], *p* = 0.003), whereas previous therapy related to osteoporosis was positively but only marginally associated with adherence (OR 1.36 [95% CI 0.98–1.88], *p* = 0.064, Table 2).

**Table 2:** Logistic regression analysis of good to very good adherence to once-daily teriparatide

	Good to very good adherence (> 75%)	Crude OR (95% CI) [*p* value]	Adjusted OR (95% CI)	*p* value
Patient-support program enrollment = yes	428/790 (54.2%)	1.27 (1.01–1.60) [0.046]	1.44 (1.04–2.00)	0.030
Patient-support program enrollment = no	221/458 (48.3%)			
Age (years)	–	–	0.99 (0.97–1.01)	0.345
Sex (male vs. female)	–	–	1.24 (0.68–2.25)	0.484
Smoking status (yes vs. no)	–	–	0.31 (0.14–0.68)	0.003
Previous therapy related to osteoporosis (yes vs. no)	–	–	1.36 (0.98–1.88)	0.064

The final model was adjusted for age (years), sex (male/female), smoking status (yes/no), and previous therapy related to osteoporosis (yes/no). Age and sex were included due to clinical importance and the other variables were identified using a stepwise backward elimination method

*CI* confidence interval, *OR* odds ratio

### Persistence with once-daily teriparatide

**Fig. 1:**
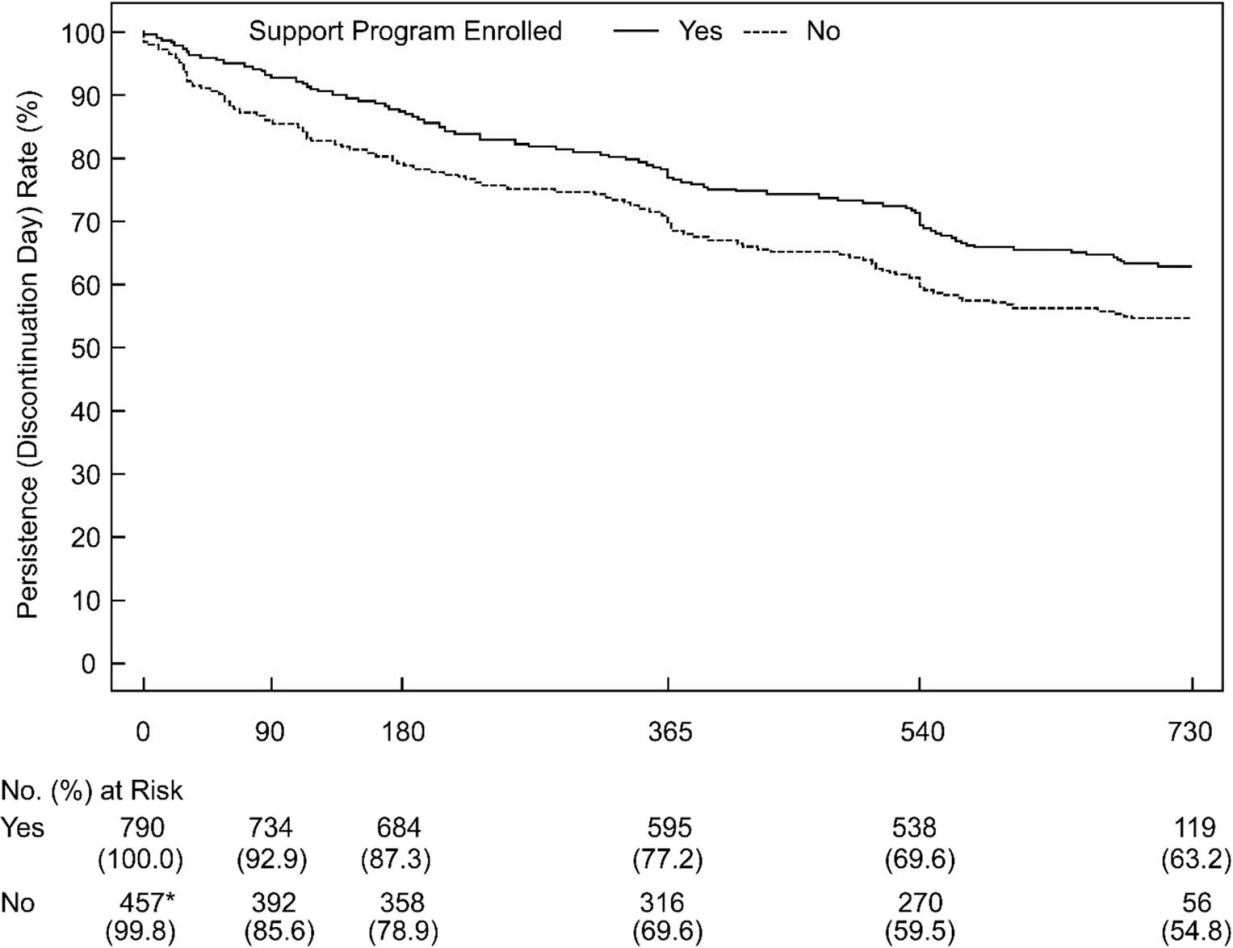
Kaplan-Meier survival curve for persistence rates with once-daily teriparatide in patients enrolled (yes) and not enrolled (no) in the patient-support program during the 24-month observation period. *One patient in the no group did not have data for initiation of teriparatide administration

We observed that persistence rates with once-daily teriparatide appeared higher for patients enrolled in the patient-support program than those who did not enroll (Fig. 1). The higher rate of persistence with teriparatide for enrolled patients was apparent within 2 to 3 months of beginning treatment and was maintained up to 24 months. Persistence rates for enrolled vs. not enrolled patients were 77.2% (95% CI 74.1–80.0%) vs. 69.6% (95% CI 65.1–73.6%) at 12 months and 63.2% (95% CI 59.6–66.5%) vs. 54.8% (95% CI 50.0–59.3%) at 24 months. The patient population at 18 months decreased to that at exactly 24 months; this was because the 24-month visit had a 30-day allowance window during which investigators could indicate that patients had completed 24 months prior to the patient completing exactly 24 months of treatment.

## Discussion

Findings from this secondary analysis suggest that patient-support programs have beneficial effects on adherence to once-daily teriparatide. Although the overall percentage of enrolled patients with good to very good (> 75%) adherence in both groups was suboptimal, higher rates of good to very good (> 75%) adherence and a significantly greater likelihood of adherence were reported in enrolled patients compared with those who did not enroll (54.2 vs. 48.3%; OR = 1.44). In addition, it was observed that persistence at 12 months (77.2%) and 24 months (63.2%) was higher among enrolled patients compared with those who did not enroll (69.6 and 54.8% at 12 and 24 months, respectively) (Fig. 1). This observation may indicate that early patient interaction and communication via the program during the initiation period is key to motivating patients to maintain persistence. In turn, the availability of an osteoporosis liaison service may be one of the beneficial options for facilitating this early interaction at the initiation of a new treatment. The key finding of a decreased likelihood of subsequent fracture after 6 months of teriparatide treatment from the primary observational study upon which this secondary analysis is based reinforces the importance of persistence [18]. Patients who discontinue within 6 months would not expect to gain this benefit from teriparatide, and so support during treatment introduction is one of the critical factors to achieving effectiveness.

Benefits on adherence were not assessed or reported in other studies of patient-support programs involving teriparatide [14–16]. Despite this, the proportion of patients with high adherence (MPR > 80%) was 48% at 24 months in a medical claims database study in the USA [11], which, despite differences in the definition of good vs. high adherence, supports our finding for patients who did not enroll in the program. In addition, our findings extend those reported in two separate studies of medical and pharmacy claims databases in Japan. The proportion of patients with high adherence (MPR > 80%) in these two studies was 61% at 12 months [21] and 56% at 18 months [12] of teriparatide treatment.

Our findings of a difference in persistence between patients who were enrolled and those who were not enrolled should take into account the descriptive nature of the analyses and that the two groups were not matched for baseline characteristics. However, benefits related to persistence have been reported in other studies of patient-support programs involving teriparatide treatment [14–16]. Spanish patients (*N* = 23,069) with severe osteoporosis, who were enrolled in a program involving regular phone contact with trained nurses designed to help them administer teriparatide correctly, had a persistence rate of 64.1% at 24 months [14]. An Italian study found that the persistence rate at 18 months among those enrolled in a support program (85.6%, *N* = 382) was 8.2% higher than that among non-enrolled patients (persistence = 77.4%, *N* = 398) [16]. Finally, a large study of French patients (*N* = 5413) also reported high persistence (81.5% over 15 months) among those enrolled in a patient-support program [15]. The lower persistence rate noted among Japanese patients in our study compared with those noted in the Italian and French studies may relate to the intensity of follow-up and injection training offered, particularly at the start of the program. For example, in the French study, up to five phone calls could be made during the first month to train patients in self-injection and evaluate their ability, with further home-based training offered for patients experiencing difficulty [15]. Similarly, patients enrolled in the Italian study were phoned weekly during the first month [16]. In contrast, Japanese patients enrolled in this analysis of the support program were called at the patient’s request during the first 6 months, in addition to receiving a monthly calendar containing a daily injection checklist and certificates of recognition for treatment milestones. Our findings, however, were similar to those of a Canadian patient-support program among 1676 patients administering 6-monthly injections of denosumab [22], in which 59.1% of patients persisted with denosumab at 24 months. In this Canadian study, follow-up by phone or email continued at 6-monthly intervals over the course of therapy.

Apart from higher adherence, bivariate analysis found several differences in baseline demographic and clinical characteristics among patients with patient-support program enrollment. Specifically, there were statistically greater proportions of females, patients with a family history of fracture, prevalent non-vertebral fractures, less severe back pain, and previous use of bisphosphonates and active vitamin D_3_ among those enrolled in the patient-support program. The fact that patients who enrolled in the program had the highest rate of prevalent non-vertebral fractures suggests that this was possibly a key motivator for enrollment. Alternatively, patients who did not enroll may have required less support given that their rate of good to very good adherence was higher than that with an unknown enrollment status (48.3 vs. 28.7%, respectively). Patients who did not enroll had significantly higher mean back pain scores at baseline (55.8 ± 30.9) compared with those who did enroll (46.9 ± 30.6). This may relate to the lower use of previous osteoporosis medication among patients who did not enroll and possible use of alternative methods to improve their back pain. Ultimately, however, it is difficult to make conclusions based on the differences between the enrollment groups in terms of back pain and the prevalence of non-vertebral fractures for two reasons: a clear pathophysiological mechanism for pain (e.g., from spinal cord compression) has not been confirmed in patients with fractures and the manifestations of back pain are variable in this population [18]. Our finding that a higher proportion of females tended to enroll in the patient-support program in Japan may relate to higher anxiety among females about their higher fracture risk and such programs being less attractive to men, although this is speculative. This finding also contrasts with that of Tamone et al. [16], who found that a lower proportion of females enrolled in an educational and phone call follow-up program (86.9% vs. did not enroll 97.7%) to help Italian patients with severe osteoporosis administer once-daily injections of teriparatide. However, this difference was found not to influence persistence rates [16]. Finally, from multivariate analysis using a backward elimination method, the independent variables in addition to the patient-support program associated with good to very good adherence among Japanese patients were non-smoking status and previous osteoporosis medication use. Other studies of patient-support programs with teriparatide did not assess these associations with treatment adherence [14–16]. However, the Canadian study of denosumab found several factors significantly associated with higher 24-month denosumab persistence, including private medication insurance, age 75 or above, previous osteoporosis medication use, and previous fracture [22].

Several limitations of this study need to be addressed. First, the investigators’ assessment of adherence was based on remaining medication and communication with patients at each visit and may not reflect actual adherence. Second, enrollment in the patient-support program was voluntary at any time during the course of treatment. As such, patients who were self-motivated and invested in maintaining good health may have been more open to enrolling in the program and more predisposed to take medications as prescribed. In addition, the definition of patient-support program enrollment in this analysis included patients who ever enrolled at any time during the 24-month observational period, leading to a potential for misclassification of enrollment status. This might explain the suboptimal rate of adherence among those who enrolled. Further research evaluating timing and duration of program enrollment is warranted. Third, although we adjusted our analyses for the major confounding factors known to contribute to the association between patient-support program and adherence, it is possible that other, unobserved confounding factors were not accounted for. Fourth, as our findings are from a cross-sectional analysis, we can only show the association between the impact of the program and adherence but cannot establish causation. Fifth, there were many missing values on patients’ records. Only 655 of 1248 (52.5%) patients had complete information on all variables, which was used for the backward elimination method as part of the multivariate analysis. We conducted a sensitivity analysis specifying variables included in the multivariate model, which increased the sample size from 655 to 1117. The adjusted OR for adherence in patients enrolled in the patient-support program according to this sensitivity analysis was 1.31 (95% CI 1.02–1.67, *p* = 0.035), which did not change the overall result. Finally, our study incorporated a large proportion of patients in an “unknown” group where enrollment status could not be determined and, although data collection for this group was attempted by physicians, it was difficult to achieve this, and so we were unable to analyze this group.

In conclusion, Japanese patients with osteoporosis who enrolled in a patient-support program were found to have better adherence to and observed better persistence with once-daily teriparatide in this secondary analysis of data conducted in a real-world clinical setting [17]. Our findings highlight the value of patient-support programs in helping patients adhere to and persist with their medication regimen, which has been shown to provide benefits in terms of clinical outcomes [10–13]. Further work would help improve adherence outcomes. Treating physicians may also find that this program provides them assistance in encouraging adherence and persistence among their patients.
